# Alkali Uptake, Release, and Speciation in Fluidized Beds Using Oxygen Carriers

**DOI:** 10.1021/acs.energyfuels.4c05523

**Published:** 2025-02-04

**Authors:** Viktor Andersson, Jan B. C. Pettersson, Thomas Allgurén, Pavleta Knutsson, Klas Andersson

**Affiliations:** † Department of Space, Earth and Environment, Division of Energy Technology, Chalmers University of Technology, Hörsalsvägen 7A, SE-412 96 Gothenburg, Sweden; ‡ Department of Chemistry and Molecular Biology, Division of Atmospheric Science, University of Gothenburg, Medicinaregatan 7B, SE-413 90 Gothenburg, Sweden; § Department of Chemistry and Chemical Engineering, Chalmers University of Technology, Kemigården 4, SE-412 96 Gothenburg, Sweden; ∥ Department of Chemical Engineering, University of Utah, Salt Lake City, Utah 84112, United States

## Abstract

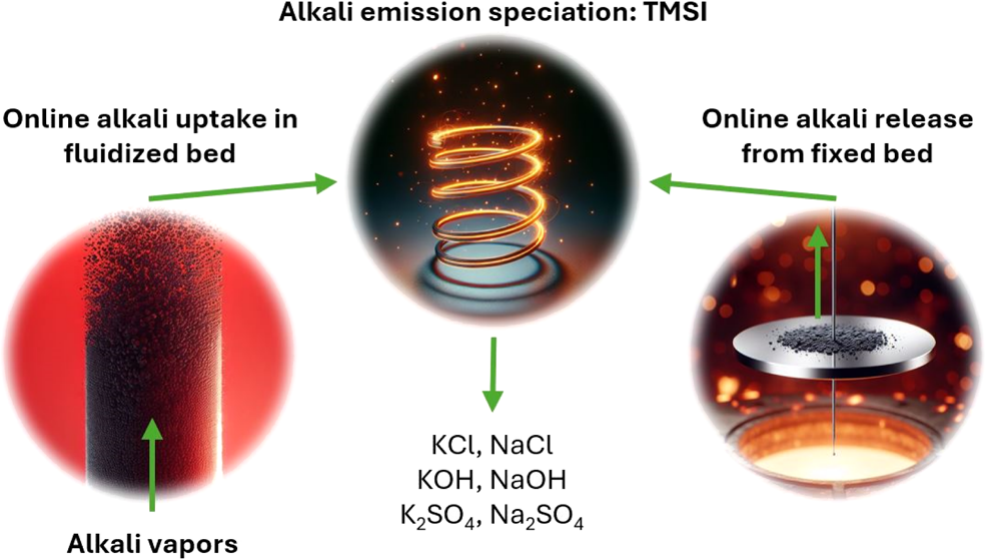

Recent advancements in combustion-related alkali chemistry have been increasingly driven by the adoption of CO_2_-neutral fuels, such as bioderived materials and waste, which often contain high amounts of alkali compounds. While alkali compounds may have catalytic effects on, e.g., fuel conversion and tar cracking, they also contribute to fluidized bed agglomeration, ash deposition, and corrosion. A thorough understanding of alkali uptake, release, and emission control is therefore crucial for scaling up and commercializing advanced fuel conversion technologies. This study presents recently developed methods for high-temperature alkali analysis, including (1) a temperature-modulated surface ionization (TMSI) technique for real-time alkali speciation, (2) a laboratory-scale reactor enabling continuous alkali vapor injection into fluidized beds with real-time monitoring of exhaust alkali emissions, and (3) a TMSI-thermogravimetric analysis (TGA) method for monitoring real-time alkali release and mass loss. The summarized results provide valuable insights into high-temperature alkali chemistry processes and their interaction with different oxygen carriers. Oxygen carriers of calcium manganite, manganese oxide, and ilmenite exhibit varying alkali uptake efficiencies based on reactor gas conditions. Ilmenite showed near-complete alkali absorption (>90% uptake of alkali chlorides), particularly in reducing conditions. Alkali speciation analysis revealed that NaCl and KCl were the main alkali species emitted during NaCl and KCl injections, with a similar trend for alkali sulfates. Ilmenite previously used as an oxygen carrier industrially releases alkali at high temperatures in both inert and oxidizing conditions. Furthermore, the TMSI method was applied to study alkali emissions during biomass pyrolysis, where KOH dominated emissions during low-temperature pyrolysis, while both KOH and NaOH were emitted from the remaining char and ash. This real-time characterization of sodium and potassium compounds offers new opportunities to optimize solid fuel conversion processes for fuels such as low-grade biomass, waste, and coal.

## 1 Introduction

As societies strive to reduce greenhouse gas emissions, efforts are increasingly focused on lowering the emissions of CO_2_ from fossil fuels. This may be achieved by introducing fuels that are considered partly or fully CO_2_-neutral. Energy sources such as biomass, which over time balance CO_2_ absorption and emission during growth and combustion, are often regarded as CO_2_-neutral. Waste streams, such as industrial or municipal solid waste, are typically considered to be partly CO_2_-neutral due to their fossil content. Another way to drastically reduce CO_2_ emissions from fossil fuel-based industries is by utilizing carbon capture and storage (CCS) technologies.^1^ However, reducing CO_2_ emissions is probably not enough to reach the goals set by the Paris Agreement.^2^ Instead, IPCC suggests implementation of negative emission technologies.^3^ One cost-effective option is to combine CO_2_-neutral fuels with CCS to achieve net negative CO_2_ emissions.^4^

Compared to fossil fuels, biomass and wastes generally contain more water, possess a lower heating value, and contain significant amounts of inorganic compounds.^5^ Among the most problematic aspects of using biomass fuels is the high content of alkali metal compounds, primarily containing K and Na, which are readily released during the conversion process.^6^ Alkali compounds are known for their unique chemical properties that can either catalyze or inhibit chemical reactions depending on the specific application and conditions. Although studies show that alkali compounds may have positive catalytic effects on char reactivity,^7,8^ biomass gasification rates, and tar cracking,^9,10^ their presence in combustion processes is often related with material degradation issues. Some of the most common issues include agglomeration of fluidized bed material and alkali-induced fouling and corrosion of heat exchanger equipment.^5^ Conclusively, alkali compounds and their release characteristics have significant impacts on solid fuel conversion.

For fuel conversion technologies based on fluidized beds, it is important to know not only how the alkali species are released from the fuel but also how they interact with the bed material. How the alkali interacts with bed material may vary between applications and the type of material. In fluidized bed applications aimed at reducing CO_2_ emissions, the use of active bed materials has attracted considerable interest. One notable group of active bed materials is oxygen carriers (OC), which can be applied across several technologies, including chemical looping combustion (CLC), chemical looping gasification (CLG), and oxygen carrier-aided combustion (OCAC).

In CLC, a variety of fuels including biomass, can be thermally converted with subsequent CO_2_ capture and storage, enabling net negative CO_2_ emissions.^11,12^ The technology utilizes two interconnected circulating fluidized bed reactors: one (referred to as the air reactor) with oxidizing conditions and one (referred to as the fuel reactor) with reducing conditions. The oxygen carrier particles facilitate controlled combustion by transporting oxygen from the air reactor to the fuel reactor. The CLC process provides the same net energy release as conventional combustion, but with the added benefit of low-cost CO_2_ sequestration.^13,14^ While CLC aims for complete fuel conversion to form products of CO_2_ and H_2_O, partial combustion is used in the CLG processes. The goal of partial fuel conversion in the CLG gasifier is to form products rich in, e.g., CH_4_, CO, and H_2_, which can be used as fuel or as raw materials in other processes.^15,16^ Beyond CLC and CLG, oxygen carriers can also be used to enhance performance in conventional fluidized bed boilers through OCAC. Here, the fuel conversion is restricted to one fluidized bed reactor, where the particles undergo oxidation and reduction in oxygen-rich and fuel-rich parts of the boiler, respectively.^17−19^ The benefits of using an OC bed in OCAC, over the typical quartz sand bed in conventional fluidized bed boilers, are increased combustion efficiency and more even temperature distribution within the bed. This enables an increased fuel load, reduced need for excess oxygen, and overall lower formation of harmful NO_*x*_ and CO emissions.^17^

Monitoring the alkali behavior during fuel conversion is essential, not only to ensure efficient operation of the conversion technologies but also to understand the chemical processes taking place in the reactor, validation of modeling results, and development of numerical simulation models. The complex behavior of alkali compounds makes in-depth studies of gaseous alkali processes challenging. In large-scale facilities, alkali studies typically depend on extractive gas sampling,^20,21^ where issues like deposition, condensation, and chemical transformations on surrounding surfaces can affect the alkali behavior. Using laboratory-scale reactors addresses many of these issues by offering more controlled process conditions, such as temperature, pressure, and gas composition. However, the high surface-to-volume ratios and elevated wall temperatures in lab reactors may influence the alkali behavior.^22^ Alkali studies generally employ stainless-steel reactors,^23−25^ operating in alternating oxidizing and reducing atmospheres. These shifting atmospheres may alter both the chemical form of gaseous alkali and the behavior of the steel walls, thereby influencing interactions of alkali with the walls. Studies show that interactions between alkali and a fluidized bed can be obscured when the surrounding reactor walls cause significant effects.^22,26^ Therefore, high-quality lab reactors are needed to allow for more accurate tracking of alkali release, transformation, and deposition, ultimately supporting the development of effective industrial-scale systems.

Besides well-functioning reactor systems, reliable and adequate alkali monitoring methods are needed to monitor the behavior of alkalis in thermal conversion applications. Various techniques have been applied to study alkali in different fuel-converting processes, categorized into offline and online methods. Offline methods, like scanning electron microscopy coupled energy-dispersive X-ray spectroscopy (SEM-EDX),^27,28^ X-ray diffraction (XRD),^29^ and X-ray photoelectron spectroscopy (XPS),^19^ analyze samples that have been extracted from the process. Although detailed information about the alkali distribution in the samples is provided, they cannot offer real-time or dynamic information. Online methods offer real-time insights using in situ measurements or techniques that rely on continuous gas extraction.^30^ In situ techniques may employ nonintrusive optical diagnostic methods, such as laser-induced fluorescence^31^ or absorption spectroscopy.^32,33^ Some of the most recognized optical techniques include in situ alkali chlorine monitor (IACM),^34^ tunable diode laser atomic absorption spectroscopy (TDLAS)^32,35^ and collinear photofragmentation and atomic absorption spectroscopy (CPFAAS).^33^ Extractive gas sampling techniques utilize various ionization approaches, with techniques including molecular beam mass spectrometry (MBMS),^36^ inductively coupled plasma mass spectrometry (ICP-MS),^37,38^ or surface ionization (SI).^21,39^

While in situ optical techniques provide detailed alkali information with high spatial and temporal resolution, they are usually limited to potassium-based alkali species (atomic K, KOH, and KCl).^6,35^ In addition, optical techniques and mass spectrometry techniques often come with a high investment cost. In comparison, the surface ionization detector (SID) is a portable, low-cost device that measures alkali concentrations in a sample gas with high time resolution.^21^ Previously, SID measurements have been limited by reporting total alkali concentration (K and Na) without further speciation.^40−42^ It is of interest to specify different alkali compounds in greater detail, since potassium and sodium often coexist in biomass and their ionic form may impact the fuel conversion processes as well as sulfation and chlorination processes.^43^ As such, a SID was used for online alkali monitoring in this work, presenting the recently developed method to speciate different alkali compounds in a sample gas.^44^

This paper summarizes and discusses a series of related developments that are of high relevance to the role of alkali compounds in energy conversion applications. The work presents recently developed laboratory reactor systems to monitor the alkali dynamics between different alkali salt compounds and different OC materials.^45,46^ In addition, the work summarizes research on gas–solid interactions between alkali and OC materials under conditions relevant for chemical looping applications.^26,47,48^ This includes studying the uptake and release of gaseous alkali compounds from OC materials under a fluidized bed or fixed bed conditions. The development of new measurement techniques and reactors opens up new possibilities for detailed alkali studies at high temperatures.

## 2 Experimental Section

This paper presents a summary of previous investigations into alkali interactions with various OC materials, studying alkali uptake and release from the materials in different reactor systems. Experiments include (i) continuous alkali gas injection into fluidized beds to characterize alkali uptake and (ii) alkali release from bed particles used for biomass combustion in an industrial application. The work employs two different fluidized bed batch reactors to study alkali uptake and a thermogravimetric analyzer (TGA) to study alkali release.

The summary also presents recent advancements in method development for detailed alkali studies in high-temperature processes. This includes the design of new reactor systems, the development of the alkali measurement system (SID) that allows alkali emission speciation, and the integration of SID with a TGA.

Overall, the studies included in this work are presented in Table 1. In addition to the references listed in Table 1, original results from emission speciation of alkali release from used ilmenite particles are presented in Section 3.4 (Figure 12).

**Table 1:** List of Studies Included in the Paper

online alkali uptake characterization			
type of study	application	alkali injection	alkali measurement
alkali–wall interactions in a typical laboratory-scale reactor^22^	reactor I	KCl	total alkali concentration
alkali interactions with calcium manganite particles^26^	fluidized bed reactor I	KCl	total alkali concentration
design and application of novel laboratory reactor for alkali studies^45^	reactor II	KCl, KOH	total alkali concentration
alkali interactions with calcium manganite, ilmenite, and manganese oxide particles^47^	fluidized bed reactor II	KCl, KOH, K_2_SO_4_, NaCl, NaOH, Na_2_SO_4_	total alkali concentration and alkali emission speciation

online alkali release characterization			
type of study	application	sample	alkali measurement
method to monitor alkali release and mass loss from samples^46^	TGA	biomass	total alkali concentration
		ilmenite	
kinetic study of alkali release from used ilmenite particles^48^	TGA	ilmenite	total alkali concentration

speciation of gaseous alkali emissions		
type of study	application	included alkali species
method for online speciation of gaseous alkali compounds^44^	TGA	KCl, KOH, K_2_CO_3_, K_2_SO_4_, NaCl, NaOH, Na_2_CO_3_, Na_2_SO_4_
alkali interactions with calcium manganite, ilmenite, and manganese oxide particles^47^	reactor II	KCl, KOH, K_2_CO_3_, K_2_SO_4_, NaCl, NaOH, Na_2_CO_3_, Na_2_SO_4_

### 2.1 Alkali Uptake in Fluidized Bed Reactors

**Figure 1:**
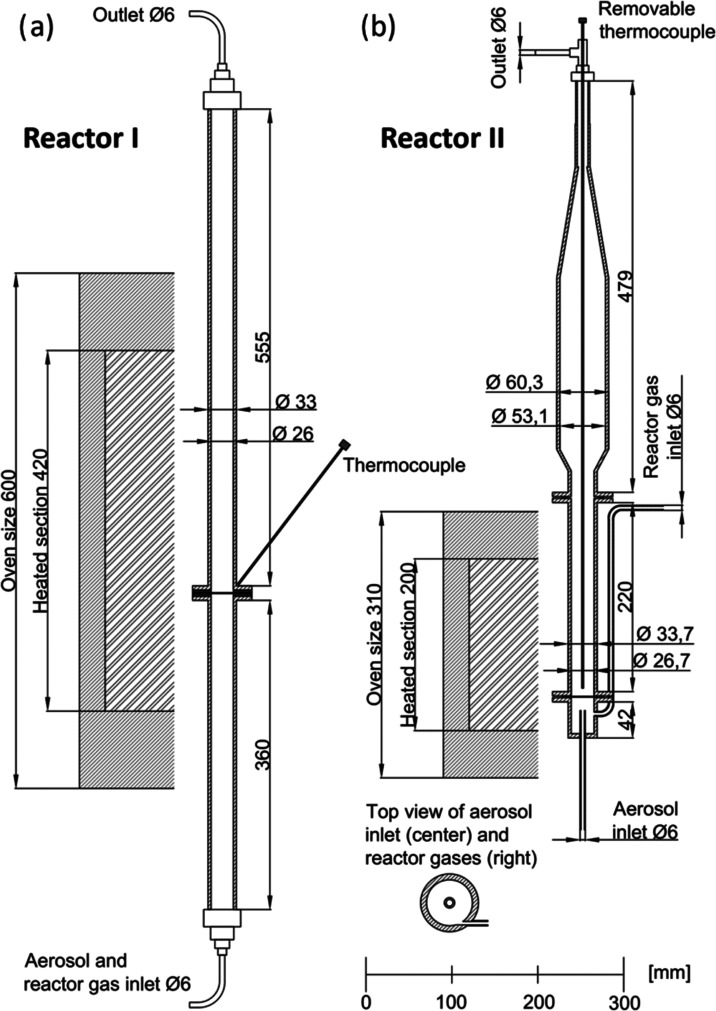
Schematic overview of the two reactors used in this study. (a) Reactor I—stainless-steel alloy 304 and (b) Reactor II—Kanthal APMT steel. Given dimensions for the respective parts of the reactors are given in mm.

Two different laboratory-scale batch reactors (illustrated in Figure 1) were used to study alkali uptake characteristics in fluidized beds of OC materials. Reactor I was a vertical tube made of stainless-steel alloy 304, heated by an electrical furnace (dimensions are shown in Figure 1a). It used a stainless-steel alloy 316 perforated plate as a particle filter and gas distributor. However, a significant limitation of Reactor I was the presence of extended hot regions above and below the fluidized bed. These regions led to unwanted interactions between alkali species and the reactor walls, obscuring the interactions between alkali and the bed particles, which are central to understanding alkali uptake.^22^ This limitation highlighted the need for a reactor that could more accurately isolate and study these interactions with less interference.

To address the limitations of Reactor I, a novel laboratory reactor (reactor II in Figure 1b) was recently designed. The new design minimizes alkali losses below and above the fluidized bed, thereby ensuring that the observed interactions are representative of the intended study. The reactor design is based on a combination of CFD simulations and fundamental understanding of the behavior of alkali aerosol particles and gases at high temperatures.^45^ The reactor (dimensions in Figure 1b), constructed using corrosion-resistant Kanthal APMT^**TM**^ steel, incorporates several innovative features that enable more accurate and reliable investigations of alkali uptake in fluidized bed systems. The reactor configuration can be separated into three main sections:

(i) Gas and alkali inlet section below the bed: A narrow tube introduces cold alkali aerosol particles just before the fluidized bed, maintaining a temperature below alkali aerosol particle evaporation and thus minimizing wall losses.^49^ Reactor gases are preheated to facilitate even bed temperature and introduced tangentially into the reactor, creating a swirling motion that shields the centered aerosol flow from the walls (bottom Figure 1b).
(ii) Hot fluidized bed section: The furnace height is reduced by more than half, decreasing heated wall surface above the bed and thus alkali–wall interactions. Constructed from Kanthal APMT, Reactor II forms a stable, nonscaling α-Al_2_O_3_ surface oxide, reducing chemical reactions with alkali compared to the lower-grade stainless-steel alloy used for Reactor I.^50,51^
(iii) Ambient temperature section above the bed: A wider diameter downstream of the furnace lowers gas velocities and enhances cooling, which aids alkali aerosol nucleation and consequently alkali transportation to downstream instruments.^49^ The design also prevents fluidized bed particles from being carried away with the flow and reduces the overall surface-to-volume ratio, limiting alkali condensation onto cold surfaces.

#### 2.1.1 Experimental Setup—Fluidized Bed Reactors

**Figure 2:**
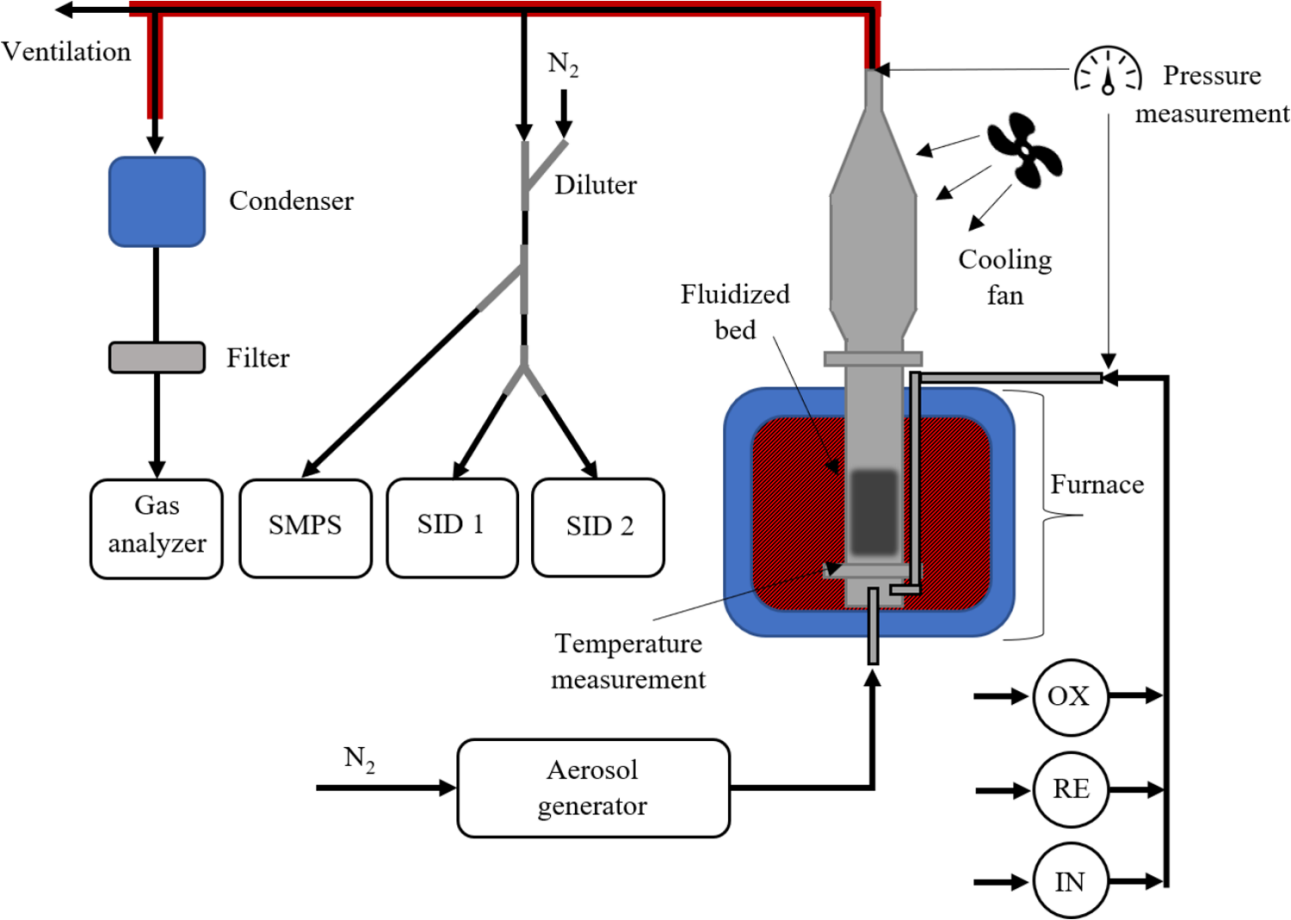
Schematic illustration of the experimental setup for the fluidized bed reactor experiments. The schematic illustrates Reactor II, externally heated by an electric furnace. An aerosol generator feeds alkali to the bottom of the reactor, and concentrations of alkali, submicrometer particles, and gases are measured in the exhaust.

The experimental setup for the fluidized bed experiments was similar for both reactors, and is illustrated in Figure 2 and explained in detail in ref (47). The reactors are placed in electrically heated furnaces where the temperature is adjusted based on temperature measurements on the lower flanges. An automated valve system regulated the flow of inert, reducing, or oxidizing gases mixed with alkali aerosol particles suspended in nitrogen and fed into the reactor from the bottom. Due to the Kelvin effect, aerosol salt particles evaporate to their molecular constituents at lower temperatures than the salt melting temperature.^52^ Consequently, the 45 nm size alkali chloride and alkali hydroxide particles used here rapidly evaporate around 500 °C, and the alkali sulfates completely evaporate below 800 °C.^22,53^ The resulting alkali gas mixture passed through a perforated plate into the bed with sufficient gas velocities to fluidize the bed. The exiting gas was directed to instruments for online monitoring of gas composition, submicron particles, and alkali concentrations.

The experimental parameters for different measurement campaigns are given in Table 2. The table includes operating temperatures, fluidized bed composition and inventory, alkali compounds and loading, and the type of measurement equipment being used in the studies. The types of alkali salt compounds were chosen based on their abundance in applications for fluidized bed conversion. Potassium is the predominant alkali release species in biomass conversion, that readily forms corrosive the compounds KCl and KOH or the less corrosive K_2_SO_4_, depending on the availability of Cl and S.^47^ In contrast, sodium compounds generally dominate the alkali release in conversion of waste, aquatic biomass, and coal fuels.^6^

**Table 2:** Experimental Parameters Used for the Fluidized Bed Experiments in Different Campaigns

paper ref	(22)	(26)	(45)	(47)
reactor	I	I	II	II
temperature (°C)	25–900	800, 850, and 900	25–900	900
bed inventory (g)	0	0, 20	0, 5, 10, 20	0, 40
bed material		calcium manganite	calcium manganite	calcium manganite, manganese oxide, ilmenite
alkali compound	KCl	KCl	KOH, KCl	KCl, KOH, K_2_SO_4_, NaCl, NaOH and Na_2_SO_4_
alkali loading (mg m^–3^)	0, 6 and 12	6 and 12	5.4	20–40
alkali flow (L min^–1^)	2	2	1	1
reactor gas flow (L min^–1^)	0.3	0.3	0.3	0.5
cooling fan	no	no	yes	yes
exhaust gas measurements	particles, gas, alkali (1 SID)	gas, alkali (1 SID)	gas, alkali (1 SID)	gas, alkali (2 SIDs)
heating of exhaust lines	no	no	no	yes

All fluidized bed experiments were conducted at temperatures between 800 and 900 °C, with additional experiments conducted in empty reactors at temperatures between 25 and 900 °C. The reactor gas environment varied to simulate different process conditions using pure N_2_, synthetic air (21% O_2_ in N_2_), or synthetic fuel gas (50% H_2_ in CO) to create inert, oxidizing, or reducing conditions. The reactor gases were mixed with the alkali flow (aerosols diluted in N_2_) before entering the fluidized beds, and the resulting inlet reactor gas concentrations can be found in refs (22,26,45,47). Exhaust gas was led through a nitrogen diluter before being fed to the instruments for particle and alkali measurements and through a cooler and particle filter to a gas analyzer. A single SID measured the total alkali concentration in the sample gas with 1 s resolution to detect total alkali concentrations in the campaigns of refs (22,26,45). The campaign described in ref (47) used an additional SID that was operated differently, facilitating alkali compound speciation. Submicron particles were measured using a scanning mobility particle sizer (SMPS) for particles between 16 and 770 nm.^22^ The same gas analyzer was used in all campaigns, measuring CO, CO_2_, H_2_, CH_4_, and O_2_ concentrations.

### 2.2 Alkali Release in Thermogravimetric Analyzer

While efficient alkali uptake by the bed is desired for reducing detrimental effects like fouling and corrosion, the fluidized bed experiments showed varying alkali uptake efficiency depending on the type of bed material being used. The experiments also showed transient alkali concentration effects in the reactor exhaust when shifting between different gas conditions, indicating that absorbed alkali may desorb from the bed particles when changing the gas environment. Therefore, a method was recently developed to characterize alkali desorption from samples such as OC bed materials, catalysts, or fuels, simultaneously monitoring alkali emissions and mass loss from fixed bed samples. Using a SID, alkali concentrations were measured online in the exhaust gas from a commercial TGA, as illustrated and explained in detail in ref (46). The development quantified alkali losses that arise from molecular diffusion in hot zones and aerosol particle losses in sampling lines between the TGA sample and the SID.^46^

This setup was used to study the release of alkali from biomass samples and OC particles previously used for industrial biomass conversion under inert or oxidizing conditions at high temperatures. Samples (0.5–20 mg) were placed on a platinum sample holder and heated to 1000 °C, where they were maintained at a constant temperature. Released alkali compounds were carried by exhaust gases, diluted with N_2_, and then measured by the SID.

### 2.3 Oxygen Carrier Particles

Three different OC materials were used for the alkali uptake studies in the fluidized bed experiments: calcium manganite, manganese oxide, and ilmenite. All materials had a particle size distribution in the range 90–250 μm.^47^ Ilmenite particles were also used for the alkali release experiments, which also included samples of woody biomass with elemental composition detailed in ref (44).

Calcium Manganite (CaMn_0.775_Ti_0.125_Mg_0.1_O_3-δ_) was made using spray-drying by VITO, Belgium, and is a perovskite-type material with Mg present as a separate phase.^54^ Manganese oxide (40 wt % Mn_3_O_4_ and 60 wt % Mg-ZrO_2_) was made by Johansson et al. using freeze granulation, with manganese oxide as the active phase and magnesium-stabilized zirconia acting as inert support.^55^ Ilmenite (FeTiO_3_), a natural ore provided by Titania A/S, mostly contains Fe and Ti, with minor amounts of Si, Mg, and other elements. Fresh ilmenite was used for alkali uptake experiments, while ilmenite with 2 wt % K and 1 wt % Na, previously used in biomass combustion in a 115 MW_*th*_ circulating fluidized bed boiler was used for alkali release experiments.^48^

### 2.4 Alkali Generation and Measurement Techniques

The alkali studies presented here use a stable, controllable alkali dosing system by introducing alkali as submicron particles suspended in a gas.^52,56,57^ These particles are generated by atomizing an alkali salt solution with pressurized nitrogen through a critical orifice, creating polydisperse droplets. The droplets are then dried in an open path diffusion dryer. The mass concentration of alkali particles is proportional to the particle size, which is controlled by changing the concentration of the liquid solution.

An SMPS measured aerosol size distributions in several of the experiments, reporting particle numbers within a size range of 16–770 nm every 120 s. Assuming spherical particles with a defined density, the SMPS provided mass concentrations which were used to calibrate the SID signal, explained in detail in ref (46).

#### 2.4.1 Surface Ionization Detector (SID)

Surface ionization has been used for alkali measurements for several decades. The concept, detailed in ref (44), relies on the thermal ionization of alkali on a hot metal surface. The degree of ionization is the ratio of positive ions to neutral atoms emitted per time unit. While most atoms favor neutral desorption, alkali metals strongly favor ion emission.^46^ On a platinum surface at 1500 K, the ionization probability exceeds 99% for K and 89% for Na.^58^ Other alkali metals like Rb, Cs, and Li also have high ionization probabilities^58^ but are less abundant and typically ignored in biomass conversion studies. Alkaline earth elements desorb as ions less readily than alkali due to higher binding energy to platinum and negligible desorption rates below 1500 K.^59^ Consequently, the method is highly selective and sensitive for detecting K and Na, making it ideal for online alkali detection in conversion processes of biomass or waste.

**Figure 3:**
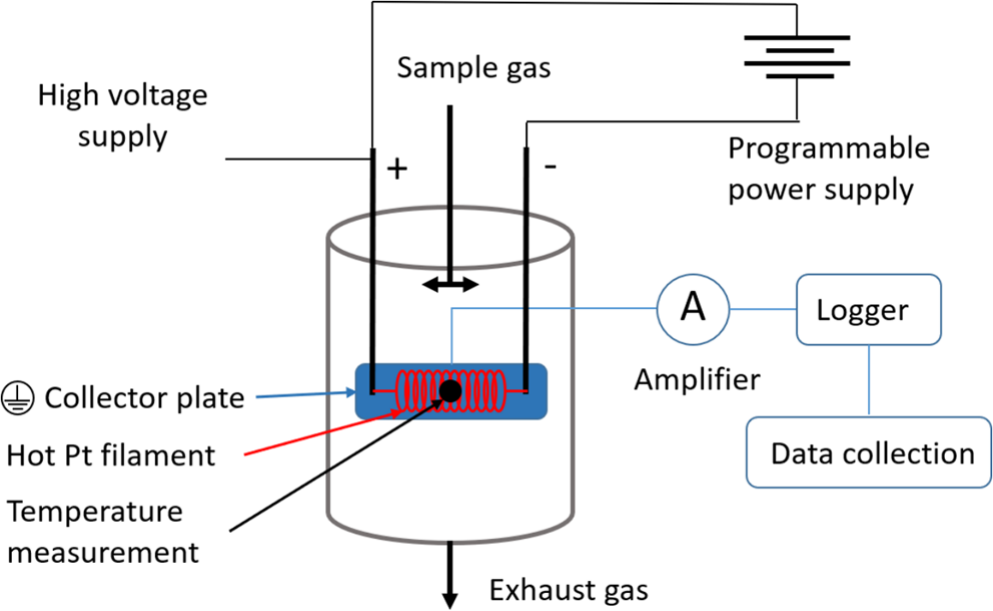
Schematic overview of the surface ionization detector (SID) including the hot platinum filament (red) and ion collector plate (blue).

The surface ionization technique has been used to measure alkali in various instruments, such as aerosol mass spectrometers and in SIDs.^20,39,60−62^ The SID, a portable and low-cost device, measures alkali concentration in gas flow.^62^ Its main components, shown in Figure 3, include a resistively heated platinum filament (red coil in Figure 3) and a closely situated metal ion collector plate (blue plate in Figure 3). When alkali-containing gas enters the SID, a fraction of the alkali will evaporate and dissociate on the hot filament before subsequently desorbing as alkali ions. The filament operates at a positive potential, forcing alkali ions to diffuse to the grounded collector plate, where the impact induces a current that is proportional to the alkali mass concentration in the sample gas.^44,63^

The current measured with the SID was transformed to an alkali mass concentration by a separate calibration experiment. In the calibration experiments, alkali aerosol particles were fed to the SID and SMPS in parallel while changing the alkali concentration. This results in near-linear relationships between the SID signal and the alkali mass concentration determined by the SMPS (for example, of calibration, see the calibration curve for KCl in ref (46)).

#### 2.4.2 Temperature-Modulated Surface Ionization (TMSI)

Previous studies have operated the SID at a constant filament temperature to measure the total alkali concentrations (K and Na) from high-temperature processes without further alkali compound speciation.^21,39,64−68^ Discrimination between K and Na compounds may be possible due to differences in their aerosol evaporation characteristics and desorption kinetics from the hot platinum filament. Based on this, a novel method, temperature-modulated surface ionization (TMSI), has been recently developed for online alkali speciation.^44^ It involves rapid shifts between filament temperatures while monitoring the alkali ion current.

Sodium atoms bind more strongly to platinum surfaces than potassium, resulting in lower desorption rate coefficients in comparison.^57,63^ Thus, at a given temperature and alkali flux, Na concentration on the platinum surface will be higher than K.^44,57^ In addition, according to the Arrhenius equation, lower temperatures decrease desorption rate coefficients.^44^ Thus, lowering the filament temperature increases the concentration of adsorbed alkali if the alkali flux remains constant. The principle of discriminating between Na and K based on their desorption kinetics is presented in ref (44), showing calculated fluxes of Na and K from a platinum filament at surface temperatures of 570 and 1118 °C. Speciation of Na and K can be made based on the distinct flux levels at each temperature and the transient flux changes during rapid temperature modulation.

The desorption model assumes that alkali compounds enter the SID in a molecular form. However, in recent applications where the SID is used to monitor alkali from conversion processes, the alkali has been in the form of submicron particles suspended in the sample gas.^21,39^ This introduces complexity but also provides an opportunity to distinguish between various alkali salts. Evaporation rates as a function of temperature differ between various salt particles, which consequently influence their specific behavior near the hot filament.^52^ For instance, aerosol particles of KCl and K_2_SO_4_ rapidly evaporate at temperatures above 500 and 800 °C, respectively,^52^ leading to markedly different behavior near the hot filament.

Based on this, a method of temperature modulation of the platinum filament was developed to speciate the alkali compounds in a sample gas. This was achieved by injecting a flow of different alkali aerosol particles into the SID during periodic shifts in platinum filament temperature while monitoring the produced ion current. The signal intensity for each type of alkali salt displays a unique dependence on filament temperature, which enabled speciation of the alkali emissions from reactor experiments.

## 3 Results and Discussion

This work presents the recent development of new methods and reactor systems and their use in alkali uptake and release investigations in high-temperature processes. This section begins with a description of the recently developed TMSI method for characterizing gaseous alkali compounds. It then addresses challenges in high-temperature alkali studies and presents the application of a novel fluidized bed reactor optimized for studying gaseous alkali interactions with bed particles. Thereafter, alkali uptake characteristics by various OC materials under oxidizing, reducing, and inert conditions are summarized. Lastly, alkali release characteristics from OC particles used in commercial biomass boilers are described, followed by an alkali emission analysis from woody biomass conversion.

### 3.1 Alkali Salt Speciation

The TMSI method enables alkali speciation based on differences in aerosol evaporation characteristics as a function of temperature for different alkali salts combined with the desorption kinetics of alkali from the hot platinum filament. Experiments were carried out by injecting alkali aerosol particles of either KCl, KOH, K_2_CO_3_, K_2_SO_4_, NaCl, NaOH, Na_2_CO_3_, or Na_2_SO_4_ to the SID during periodic shifts between low and high filament temperatures while measuring the produced ion current. Steady-state alkali signal intensities at temperatures ranging from 450 to 1000 °C were calculated, showing a unique dependence on filament temperature for each salt.^44^ Based on these results, a procedure was selected where the filament temperature is repeatedly changed between three temperatures: 550–1100–800–1100 °C, with 30 s duration at each temperature.

**Figure 4:**
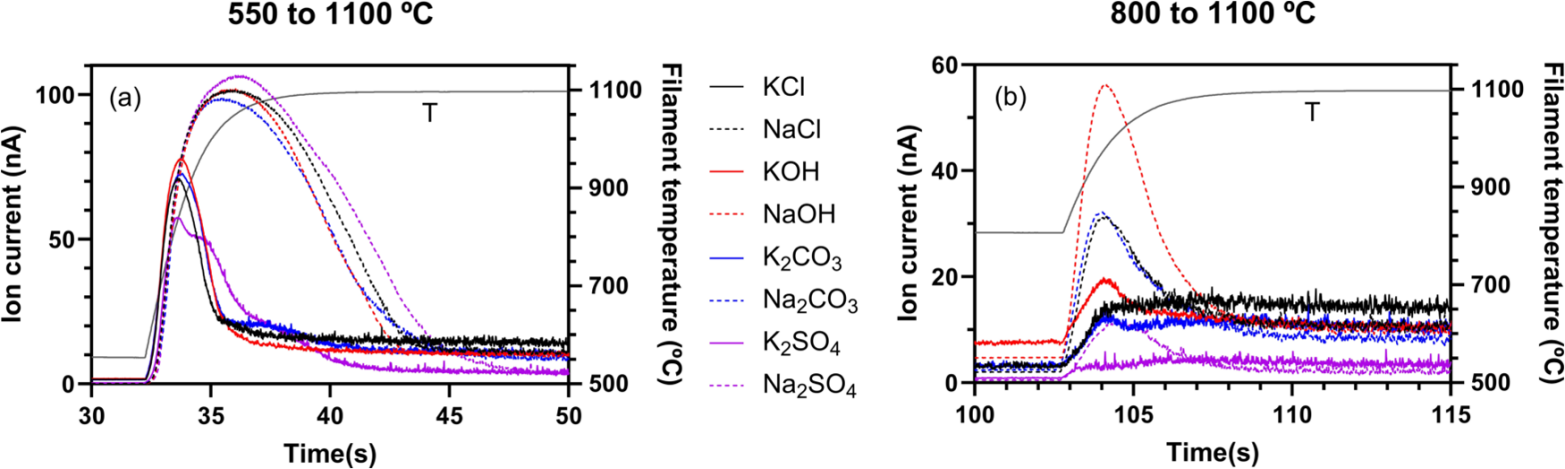
Transient change in ion current as the filament temperature is increased from (a) 550 to 1100 °C and (b) 800 to 1100 °C, with constant injection of KCl, NaCl, KOH, NaOH, K_2_CO_3_, Na_2_CO_3_, K_2_SO_4_, and Na_2_SO_4_.

By comparing the generated ion currents in steady state at the three filament temperatures, it was possible to distinguish pure alkali salts but not separate salts in a mixture.^44^ To improve selectivity, transient ion signals generated during the transition from low to high temperature were incorporated into the analysis; see Figure 4. Increasing the filament temperature from 550 to 1100 °C (Figure 4a) creates transient peaks in the alkali signal for all salts, with Na salts showing peaks significantly larger than those of K salts. For potassium salts during the shift between 800 and 1100 °C (Figure 4b), only KOH shows a clear peak, while K_2_CO_3_ generates a minor peak. In contrast, all sodium salts produce notable peaks, with the order of magnitudes being NaOH > Na_2_CO_3_ > NaCl > Na_2_SO_4_.

**Figure 5:**
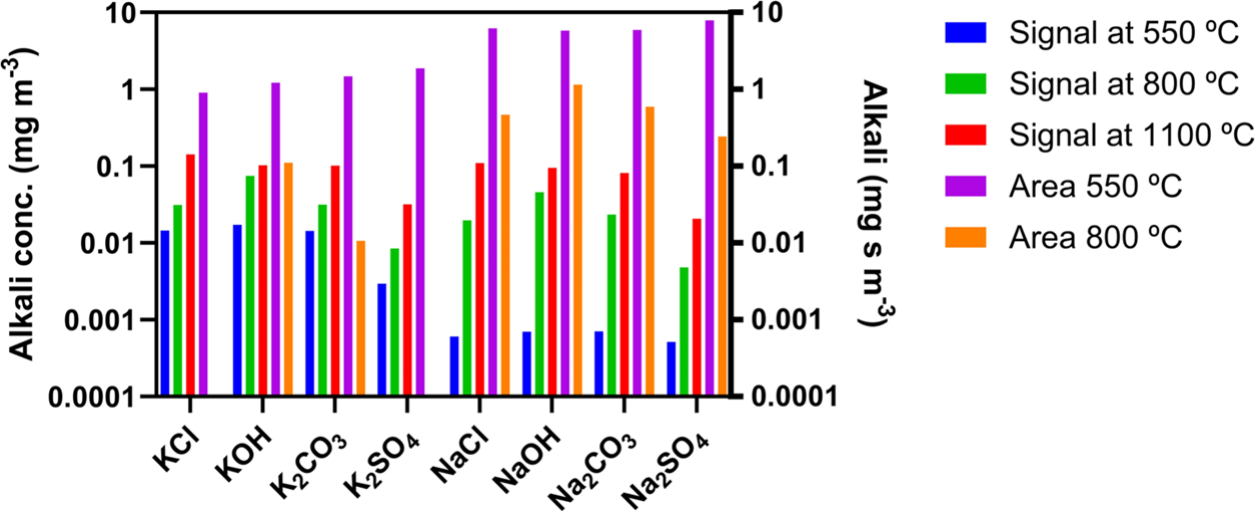
SID results from periodic temperature modulation during constant alkali injection, showing steady-state alkali signals at 550 °C (blue), 800 °C (green), and 1100 °C (red), and transient peak areas that arise when the filament temperature is increased from 550 to 1100 °C (purple) and from 800 to 1100 °C (orange).

While the time dependence of transient peaks can speciate different alkali salts, it complicates the online analysis.^44^ Instead, a simplified approach is applied that uses the total integrated area of each transient peak. Thus, the TMSI procedure produces five parameters every 2 min: steady-state signal intensities at 550, 800, and 1100 °C, and transient peak areas from temperature increases from 550 to 1100 °C and from 800 to 1100 °C. Figure 5 summarizes these five parameters for all of the salts in the study. Both Na and K show similar temperature dependencies when bound to the same counterion. Hydroxides show a strong signal at 800 °C, chlorides show a significant change in signal intensity between 800 and 1100 °C, and carbonates follow similar trends as Cl but with a less pronounced change. Transient peak areas differ significantly between K and Na salts and by alkali salt type. The differences between the five parameters for the eight different salts work as distinct “fingerprints” for each salt, aiding in their identification in unknown salt mixtures.

Discussions regarding specific trends observed for each salt compound, and the procedure for calculating the transient peak areas and steady-state signal intensities are presented along with detailed error estimates for pine wood pyrolysis in ref (44). The confidence in model parameters was evaluated by perturbing one parameter at a time from its best-fit value, refitting the others, and recording the effect on the sum of least-squares errors. This method, though more computationally demanding than standard statistical metrics, provides a realistic assessment of parameter constraints while accounting for interdependencies among parameters.^44^ The novel TMSI method, combined with the summarized data for pure alkali salt compounds, was used in refs (44) and (47) to determine the composition of alkali emissions in experiments with fluidized bed particles and in the pyrolysis of wood. In this paper, the TMSI method is used to determine the alkali emission composition in Figures 10, 12, and 13.

### 3.2 The Behavior of Alkali Compounds in Laboratory-Scale Reactors

**Figure 6:**
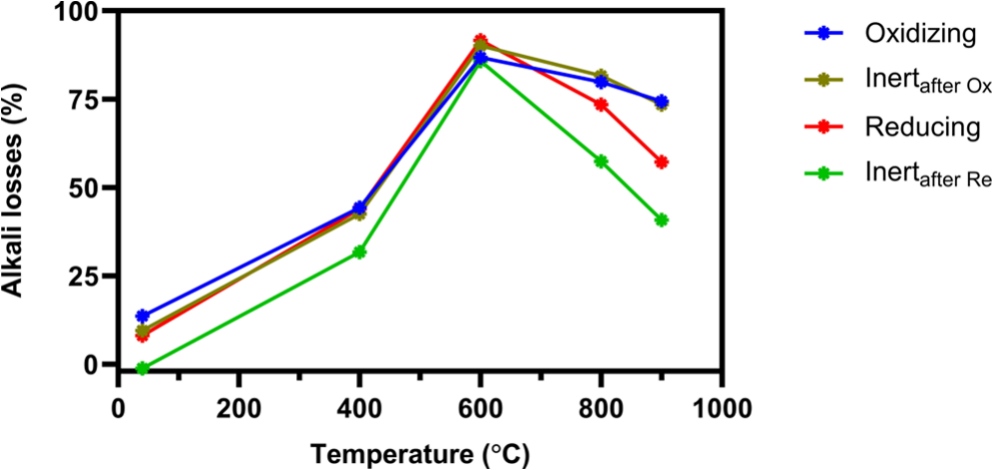
Alkali losses as a function of reactor temperature in Reactor I during the continuous injection of 6 mg m^–3^ KCl under repeated redox cycles. Each redox cycle consists of oxidizing (blue lines), inert (dark green lines), reducing (red lines), and again inert (bright green lines) conditions.

Laboratory-scale reactors are crucial for in-depth process studies, but their smaller size compared to pilot- or industrial-scale applications can pose significant challenges.^22^ The influence of reactor wall processes on alkali studies was demonstrated using an empty laboratory-scale fluidized bed reactor which is considered a typical setup.^22^ Experiments with a constant flow of KCl aerosol particles to Reactor I (Figure 1a) at temperatures between 25 and 900 °C were conducted under recurring stages of: inert, reducing, inert, and finally oxidizing conditions. Figure 6 shows the temperature dependence of alkali losses in the reactor system in each gas environment (i.e., the difference between injected alkali and the alkali concentration measured in the reactor exhaust). The two inert conditions, one following the reducing stage and the other following the oxidizing stage, are displayed in bright green and dark green colors, respectively.

Figure 6 shows minor alkali losses to the inner reactor walls at room temperature. An increase in losses is observed at 400 °C, which likely occurs due to the enhanced alkali particle diffusion at these temperatures. Increasing the temperature to 600 °C causes the KCl particles to rapidly evaporate to their molecular constituents.^22,52^ As diffusion coefficients for molecules are several orders of magnitude higher than those for aerosol particles, the alkali molecules will easily reach the reactor walls. The alkali reaching the walls can either remain stable or re-evaporate from the surface and leave the reactor with the exhaust gases.^22^ At 600 °C, most of the injected alkali will reach and remain stable at the reactor walls with alkali losses up to 92%. However, the alkali outlet concentration recovered when increasing the temperature to 800 and 900 °C, indicating enhanced re-evaporation of alkali from the wall at higher temperatures. In addition, alkali re-evaporation from the walls also depends on the gas composition in the reactor. The highest alkali outlet concentrations were observed under reducing conditions and inert conditions following the reducing stage. In contrast, oxidizing conditions and inert conditions following the oxidation stage yielded higher alkali losses.

The corresponding number and mass concentrations of submicron aerosol particles, measured by SMPS and presented in ref (22), show that at 25 and 400 °C, the aerosol particles remain intact when flowing through the reactor. At 600 °C, the mass concentration drops significantly, while the number concentration increases 10-fold, indicating the formation of newly nucleated, smaller particles. This suggests that alkali particles evaporate in the hot reactor and thereafter nucleate in the cooler downstream regions forming new aerosol particles. The findings in Figure 6 are in accordance with empirical aerosol transportation equations,^22,49^ and a semiquantitative kinetic model describing the evaporation of aerosol salt particles as a function of temperature.^52^

The influence of a fluidized bed in Reactor I (Figure 1a) was studied by injecting KCl into a fluidized bed of 20 g calcium manganite particles at temperatures between 800 and 900 °C.^26^ The results demonstrate that a fluidized bed of OC particles affects alkali behavior compared with the empty reactor experiments, and changes in gas composition led to changes in the outlet alkali level. The current setup indicates that reactor wall properties significantly influence the amount of alkali reaching a fluidized bed in Reactor I, potentially obscuring detailed interactions between bed particles and alkali.^26^ These findings of Reactor I inspired the development of a novel reactor, aiming to minimize the interactions between alkali and reactor walls above and below the fluidized bed.^45^

#### 3.2.1 Application of a Novel Laboratory Reactor

**Figure 7:**
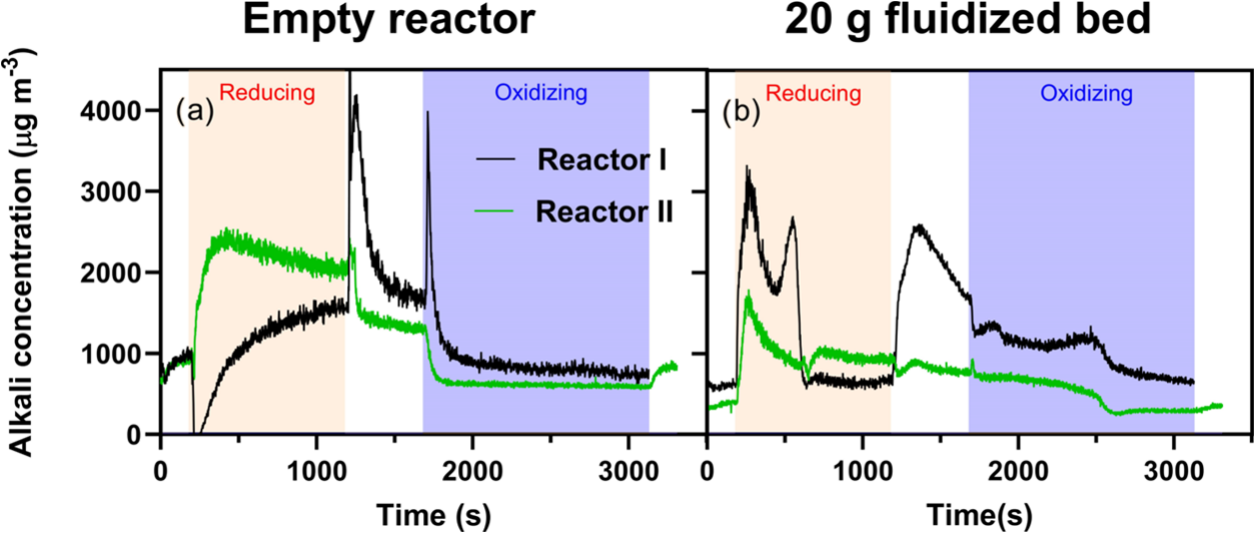
Alkali concentrations in the exhaust measured during one redox cycle at a reactor temperature of 850 °C with continuous KCl injection to (a) empty reactors and (b) reactors containing fluidized beds of 20 g of calcium manganite particles. The results include experiments using the old Reactor I (black lines) and the new Reactor II (green lines). The colored sections indicate different atmospheric conditions: blue for oxidizing, white for inert, and orange for reducing environments.

The configuration of the newly designed laboratory-scale fluidized bed reactor (Reactor II in Figure 1b) is presented in Section 2.1, and the development is described in detail in ref (45). Experiments were carried out when the reactor operated with or without the presence of a fluidized bed at different temperatures and gas conditions to evaluate the performance of the reactor.^45^Figure 7 shows a comparison in outlet alkali concentration while operating the two types of reactors (Reactors I and II) at 850 °C during constant KCl injection. The figure illustrates time-resolved data for one complete redox cycle, composed of: 180 s inert, 1000 s reducing, 500 s inert, and finally 1450 s oxidizing gas conditions.

Figure 7a shows significant differences between the reactors without the presence of a fluidized bed. Reactor I (black line) displays sharp transient peaks during transitions from reducing to inert and inert to oxidizing conditions, which are absent in Reactor II (green line). Another clear difference is observed when reducing gases are introduced, marked by a sharp drop in alkali concentration from Reactor I and a sharp increase in alkali concentration from Reactor II. Both reactors approach similar steady-state alkali concentrations toward the end of each gas stage in the redox cycle. These differences suggest that the design of Reactor II reduces transient alkali signals, which simplifies the evaluation of alkali concentration profiles in fluidized bed experiments. While changes in surface composition may affect how alkali binds to the wall surface, the less pronounced transients in Reactor II may be a consequence of a more stable wall surface layer compared to Reactor I. While the new reactor is constructed in Kanthal APMT, forming nonscaling α-Al_2_O_3_ surface oxides with excellent corrosion resistance, Reactor I is constructed in lower-grade stainless-steel alloy which mainly forms less stable Cr_2_O_3_ surface oxides. Transient alkali behavior due to differences in gas mixing and residence times between the two reactors has not been evaluated.

Figure 7b shows the corresponding results when operating each reactor with a 20 g fluidized bed of the same material. The alkali concentration profiles are notably different for the two reactors, indicating that the reactor configuration significantly impacts the results. The most pronounced difference occurs under inert conditions following the reduction phase. Reactor I shows a distinct alkali peak during this period, whereas Reactor II maintains a much steadier concentration, similar to the levels observed before and after the inert gas was introduced. Another key difference appears around the 500 s mark during the reducing phase, where Reactor I shows a second alkali peak, which is absent in Reactor II. These results indicate that Reactor II better isolates alkali interactions with the fluidized bed material, reducing interference from reactor walls.

To summarize, alkali aerosol particles remain condensed up to around 500 °C with low diffusion coefficients, making aerosol particles ideal for dosing alkali into reactors over a wide concentration range. However, at high temperatures, alkali aerosol evaporates to form alkali gases that rapidly diffuse and interact with surrounding surfaces. Therefore, the presence of hot reactor walls before or after the region of interest (e.g., fluidized beds) may obscure the alkali details that are of interest for this study (e.g., the interactions with bed particles). The novel design of Reactor II presents an improvement over Reactor I which has a design that is typically employed for these kinds of studies. The areas of hot reactor walls above and below the fluidized bed have been greatly reduced in Reactor II compared with Reactor I. This enhances the clarity of the interactions between alkali and bed material by reducing alkali losses to hot walls before and after the bed. Moreover, in the location of a fluidized bed in Reactor II, the surface area of bed particles is 1 to 2 orders of magnitude higher compared to the area of hot reactor walls.^45^ Further evidence for limited wall influence comes from experiments presented in ref (45), where the addition of merely 5 g of bed material to Reactor II qualitatively alters alkali behavior compared to an empty reactor. The same study demonstrates consistent and systematic changes in alkali behavior with variations in bed material oxidation state during redox cycles, which provide additional evidence of the improved reactor design.^45^ In addition, the lack of transients in the alkali concentration from Reactor II when changing gas composition in empty reactor experiments is an obvious advantage (Figure 7a). Together, these features substantiate the improved performance of Reactor II, even if the exact quantification of wall effects is challenging.

### 3.3 Online Characterization of Alkali Uptake by Fluidized Beds

The interaction between the bed material and alkali is of great importance for the behavior of a fluidized bed. Studies have shown that alkali in a fluidized bed can influence both fuel conversion and fluidization properties.^10,23,24^ This section discusses how the combination of reactor and measurement techniques described in this work can be used to study the uptake of different gaseous alkali compounds by three different OC fluidized bed materials that are considered state of the art in solid fuel conversion applications. Reactor II and the TMSI alkali detection method are employed to study interactions between alkali and bed material of either manganese oxide, calcium manganite, or ilmenite at 900 °C under different gas conditions.^47^ In the experiments, 40 g of the bed material was fluidized under recurring reducing, inert, and oxidizing conditions. Alkali vapors of KCl, NaCl, KOH, NaOH, K_2_SO_4_, or Na_2_SO_4_ were continuously injected into the fluidized beds while the outlet alkali and gas concentrations were monitored.

**Figure 8:**
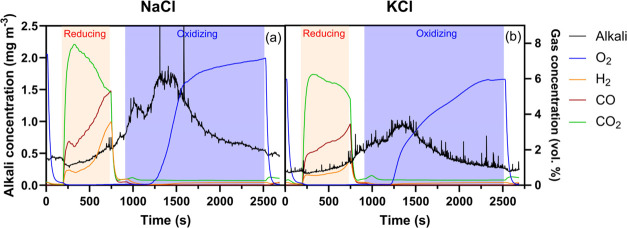
Outlet alkali (black lines) and gas concentrations (colored lines) measured from continuous injection of 20 mg m^–3^ (a) NaCl and (b) KCl into 40 g of fluidized bed of ilmenite during one redox cycle at 900 °C. The colored sections indicate different atmospheric conditions: blue for oxidizing, white for inert, and orange for reducing environments.

Figure 8 shows time-resolved data of outlet alkali and gas concentrations measured during one redox cycle while 20 mg m^–3^ NaCl or KCl were fed into a fluidized bed of 40 g ilmenite at 900 °C. The results display similar trends in alkali outlet concentration for both NaCl (Figure 8a) and KCl (Figure 8b) injection. The alkali profiles also show low alkali outlet concentrations (<2 mg m^–3^) relative to the alkali injection (20 mg m^–3^), indicating efficient alkali uptake by the ilmenite material. The gas concentrations indicate incomplete fuel conversion with about 77% of the CO being converted to CO_2_ at the beginning of the reducing phase. At the same time, only 7% of the incoming H_2_ is measured in the outlet, suggesting a more efficient hydrogen conversion process. During the oxidizing phase, full O_2_ consumption is observed for the first 300 s, after which the O_2_ concentration in the exhaust gradually rises until the bed material becomes fully oxidized, which occurs after around 1400 s.

In addition to Figure 8, data for other alkali salts and bed materials are published in ref (47). The results show that alkali uptake varies by the type of salt being injected, bed material, and the gas composition. Potassium and sodium salts of the same nature exhibit similar patterns throughout the redox cycle, implying substantial resemblances in how they interact with various types of bed materials.

**Figure 9:**
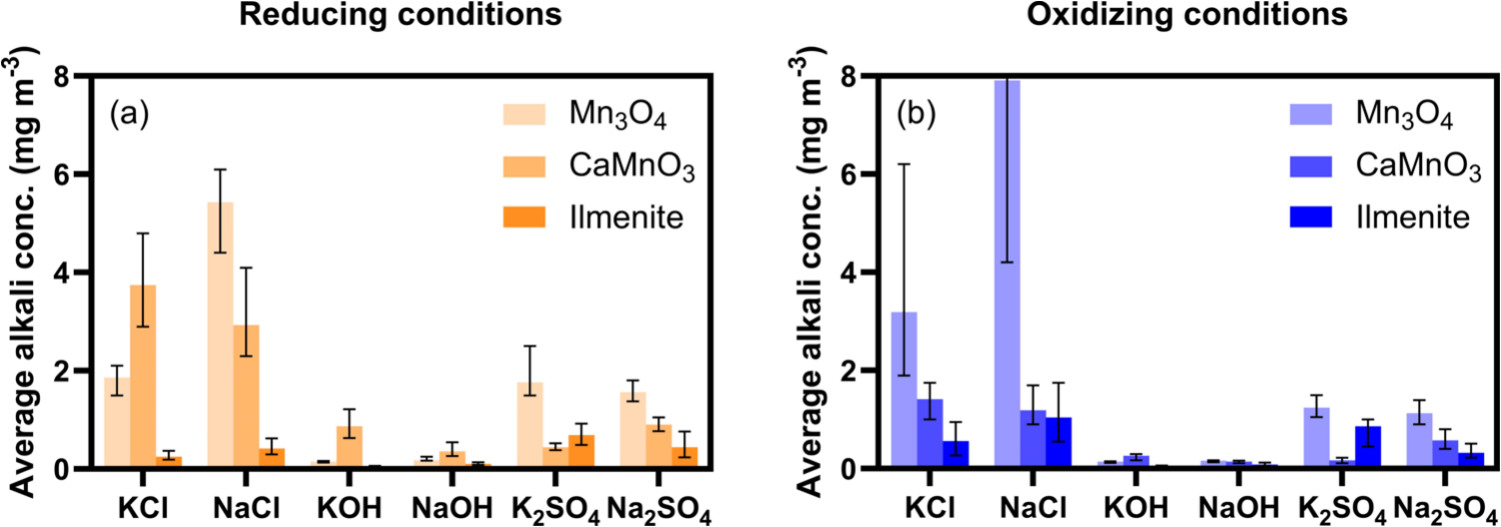
Average outlet alkali concentrations (mg m^–3^) in (a) reducing and (b) oxidizing conditions when the reactor is filled with 40 g of Mn_3_O_4_ (bright colors), CaMnO_3_ (medium bright colors), and ilmenite (dark colors) particles. Error bars indicate how much the average values deviate from the highest and lowest values of the stable alkali concentration during sampling in each gas condition. The upper error bar for NaCl injection to Mn_3_O_4_ in oxidizing conditions reaches 16.8 mg m^–3^.

Figure 9 summarizes the average alkali outlet concentrations under reducing and oxidizing conditions for all combinations of alkali salt injections and fluidized bed materials. Included error bars indicate how much the average values deviate from the highest and lowest values of the stable alkali concentration obtained under each gas condition. These results are also presented in Tables S1–S3. Although all alkali outlet concentrations remain significantly lower compared to the amount being injected, the results indicate substantial differences in alkali uptake efficiency between the different materials. Ilmenite is by far the most superior in terms of alkali uptake efficiency, followed by calcium manganite and last manganese oxide is least efficient. Depending on the type of alkali salt being injected, alkali chlorides escape the bed more easily compared to sulfates, while most of the hydroxides are lost to the bed material. Although the trends for different gas conditions are less clear, calcium manganite seems to exhibit higher alkali uptake efficiency in oxidizing conditions compared to reducing conditions, while the opposite is observed for ilmenite.

**Figure 10:**
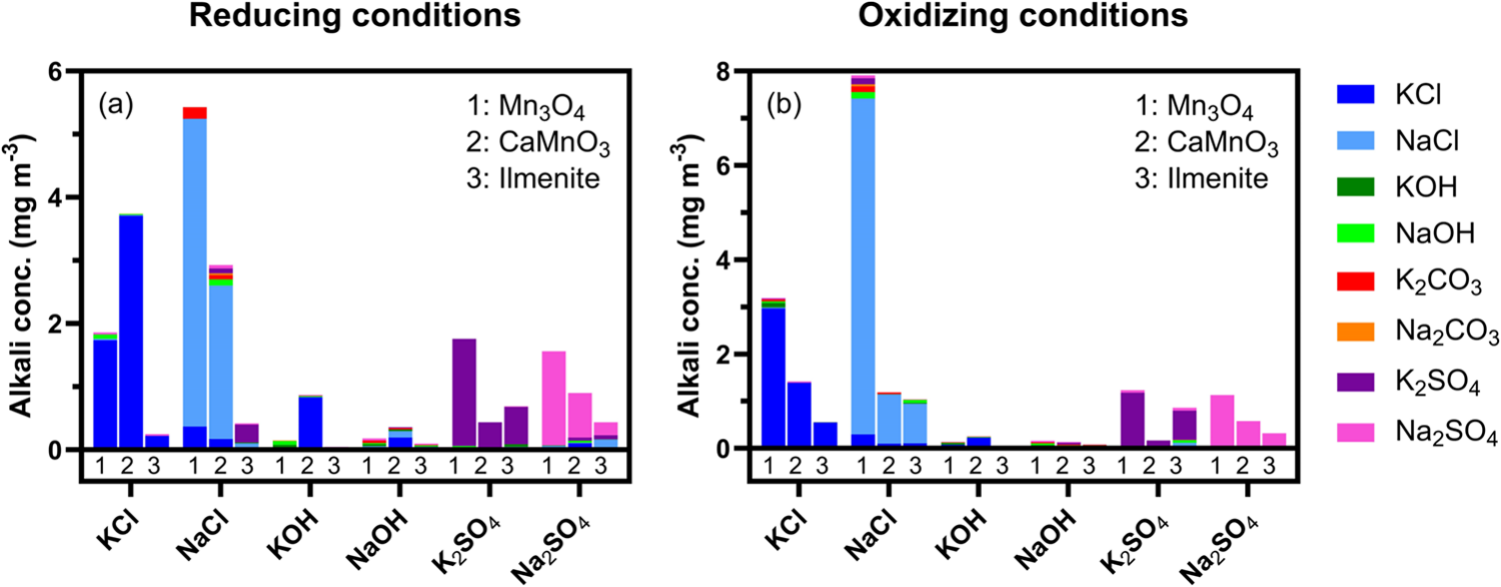
Average alkali concentration of each alkali compound leaving the reactor during (a) reducing and (b) oxidizing conditions in experiments with fluidized beds of Mn_3_O_4_, CaMnO_3_, and ilmenite. The experiments are conducted with continuous injections of KCl, NaCl, KOH, NaOH, K_2_SO_4_, or Na_2_SO_4_ aerosol.

Measurements of the alkali emissions leaving the reactor system were conducted with a SID operating according to the previously described TMSI methodology (Section 3.1). The relative contributions of different alkali species for all combinations of injected alkali salt and bed materials are presented in ref (47). Figure 10 shows the product of the average alkali outlet concentration (Figure 9) multiplied by the contribution of the different salt emissions determined by the TMSI method.^47^Figure 10a shows the speciation of average salt emissions in reducing conditions, and Figure 10b shows the corresponding in oxidizing conditions. When KCl is injected into the fluidized beds, it predominantly appears in the reactor outflow under both oxidizing and reducing conditions. This aligns with expectations, as the introduced concentration of KCl is relatively high and alkali chlorides are known to be highly volatile and a major compound released during conversion of Cl-containing fuels.^5,69,70^ The same is true during NaCl, K_2_SO_4_, and Na_2_SO_4_ injections, where NaCl, K_2_SO_4_, and Na_2_SO_4_ emerge as the primary alkali compounds in the exhaust, respectively. However, alkali hydroxide injections result in a mix of alkali compounds in the exhaust, with noticeable fractions of alkali chlorides (see KOH injection to CaMnO_3_ in Figure 10a,b, and the results in greater detail in ref (47)). However, it is essential to consider the low absolute alkali concentrations observed during hydroxide experiments, which has an impact on the precision of the analysis.^47^ In conclusion, the release of alkali hydroxides from the fluidized beds included in this study appears to be of minor importance. Instead, the release of alkali compounds may be substantially affected by the presence of other counterions including Cl^–^ and SO_4_^2–^.

#### 3.3.1 Summarizing the Experimental Results

The fluidized bed offers a large surface area for interactions between alkali and bed particles, leading to efficient alkali uptake, as illustrated by the nearly complete alkali hydroxide absorption (Figure 9). When reaching the fluidized bed, the alkali may initially adsorb to the particle surface, undergoing processes such as dissociation and diffusion. The alkali can either form strong bonds with the material that persist throughout the experiments or associate with new counterions, followed by eventual desorption.^47^ Alkali adsorption varies based on the type of alkali salts being injected and the type of bed materials (Figure 9). Sodium and potassium salts seem to behave similarly within a redox cycle (Figure 8), while available counterions (Cl-, OH-, and SO_4_^2–^) significantly affect the outcome.^47^ Alkali chlorides yield the highest alkali outlet concentrations, followed by sulfates, and these compounds also dominate the emissions in the exhaust (Figure 10). Thus, chlorine and sulfur compounds available on the particle surface likely enhance volatile compound formation, whereas alkali hydroxides are mostly absorbed with limited emissions (Figure 10), indicating reduced recombination and desorption. Alkali adsorption also depends on the chemical characteristics of the fluidized bed material under different conditions (Figure 8).^47^ Manganese oxide shows limited impact from gas composition changes, while calcium manganite shows less efficient alkali uptake in reducing conditions, likely due to oxygen deficiency affecting alkali stability.^47^ It is well-known that ilmenite effectively absorbs alkali,^27,28,39,65,71^ where a likely product is alkali titanates (e.g., KTi_8_O_16.5_^27^), consistent with low outlet alkali concentrations observed in these experiments (Figure 9). Although ilmenite is superior to the other two materials in terms of alkali uptake (Figure 9), calcium manganite and manganese oxide are more efficient in terms of fuel conversion and oxidizing efficiency.^47^

### 3.4 Online Characterization of Alkali Release

While the reactor studies presented above indicate varying degrees of alkali uptake depending on the process conditions, some of the alkali profiles showed transient effects in alkali emissions when shifting between different gas conditions within a redox cycle (Figure 8 and ref (47)). Previous alkali monitoring studies in a 100 kW chemical looping combustion (CLC) pilot also indicate that alkali can be transported from the fuel conversion reactor, to be released in the flue gases leaving the oxidizing air reactor.^39^ One potential pathway for alkali transportation is via the OC bed material. Alkali can be absorbed by the bed material in the reducing conditions of the fuel reactor before being subsequently released from the bed material when reaching the oxidizing conditions of the air reactor.

**Figure 11:**
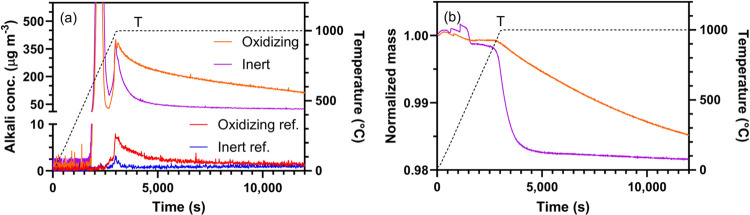
(a) Alkali desorption and (b) normalized mass loss profiles from ilmenite that were used during biomass combustion. Experiments were carried out under inert (purple) and oxidizing (orange) conditions at a heating rate of 20 °C min^–1^ followed by a 10 h period at 1000 °C. Alkali desorption from unused reference ilmenite is shown in red (oxidizing) and blue (inert).

Therefore, the TGA-SID setup was used to characterize alkali desorption from ilmenite particles previously used for industrial OCAC of biomass. The technique was deemed suitable for characterizing alkali emissions by temperature and conducting kinetic alkali release studies under isothermal conditions. The material was fully oxidized and contained approximately 2 wt % K and 1 wt % Na.^48^ The ilmenite was exposed to temperatures of up to 1000 °C under inert and oxidizing conditions. Results from 13 mg ilmenite samples (Figure 11a) show a substantial alkali desorption peak between 630 and 800 °C in both environments, likely to originate from loosely bound alkali on the particle surfaces. No substantial mass loss is observed at this stage despite the large alkali peak (Figure 11b), which demonstrates the high sensitivity of the SID. A second alkali release occurred above 900 °C and continued throughout the 1000 °C isothermal period. During the extended period with isothermal conditions at 1000 °C, both alkali release and mass loss profile decayed on a similar time scale in inert atmosphere, and the decay was approximately 5 times slower in an oxidizing atmosphere. Therefore, changes in sample properties depend on the oxygen activity of the surrounding atmosphere. The total desorption was more than twice as large under oxidizing conditions compared to inert conditions (10 and 5 wt % of the available alkali amount respectively).^48^ Almost no alkali was released from reference ilmenite (red and blue lines in Figure 11), indicating that alkali released from the used ilmenite is due to alkali introduction during combustion rather than an inherent property of the ilmenite material itself.

Material analyses revealed that significant fractions of K were bound as stable feldspars, while some additional K was found on the outer ilmenite particle surface and at the interface between the ilmenite and a Ca-rich ash layer.^48^ Na was found in association with phosphorus, ilmenite, and feldspars, with a significant concentration on the outer ilmenite particle surface. During TGA experiments, surface concentrations of K, Na, and Cl decreased, with Cl being depleted from a deeper layer under oxidizing conditions than in an inert environment.^48^

Using the TGA-SID method, Arrhenius parameters, such as activation energy (*E*_a_) and pre-exponential factor (*A*), were determined, with findings showing their dependence on both sample mass and the surrounding inert or oxidizing environment.^48^ A relatively simple, near-first-order desorption behavior was observed in the oxidizing conditions, while the alkali release mechanism appeared more complex in inert conditions. The desorption process was likely influenced by mass transfer limitations when larger sample masses were involved, affecting the observed alkali release.^48^

Additional experiments were conducted to study the alkali release from the ilmenite sample using the TMSI method to specify the alkali emissions. The time resolution of the TMSI method is 120 s, at which time the method requires relatively stable alkali concentrations to predict the alkali composition.^44^ It is, therefore, difficult to use the method to predict the alkali composition in periods with rapid changes in alkali concentration, as is the case around the first alkali peak in Figure 11a. Consequently, the TMSI method was used to predict the composition of alkali emissions from the ilmenite after the sample had reached 1000 °C where it was kept at a constant temperature for an extended period under inert conditions.

**Figure 12:**
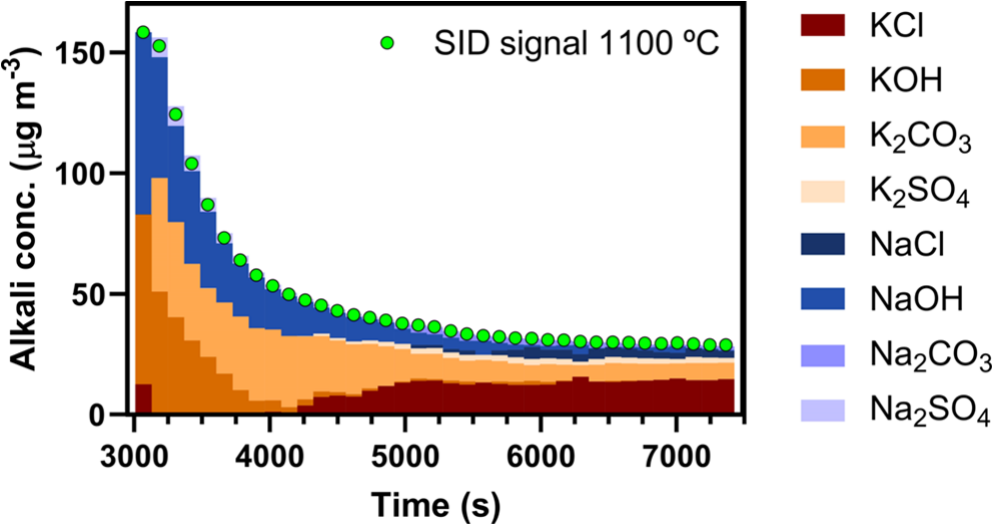
Alkali signal measured by the SID at a filament temperature of 1100 °C (green dots) during biomass conversion under inert conditions. Alkali emission composition predicted by the TMSI model (bars).

Figure 12 shows the total alkali concentration from the 1100 °C SID filament temperature operation (green dots) together with the predicted composition of the alkali emissions after the sample had reached 1000 °C (i.e., from the 3000 s time mark in Figure 11). Initially, the alkali emissions are dominated by alkali hydroxides, with similar fractions of KOH and NaOH. The alkali outlet concentration decreases significantly, along with the alkali hydroxide emissions, within the initial 1000–1500 s at the high temperature. After KOH and most of the NaOH seem to have depleted, the fraction of KCl increases and gradually starts to dominate the outlet alkali emissions.

#### 3.4.1 Alkali Emission Characterization during Wood Pyrolysis

**Figure 13:**
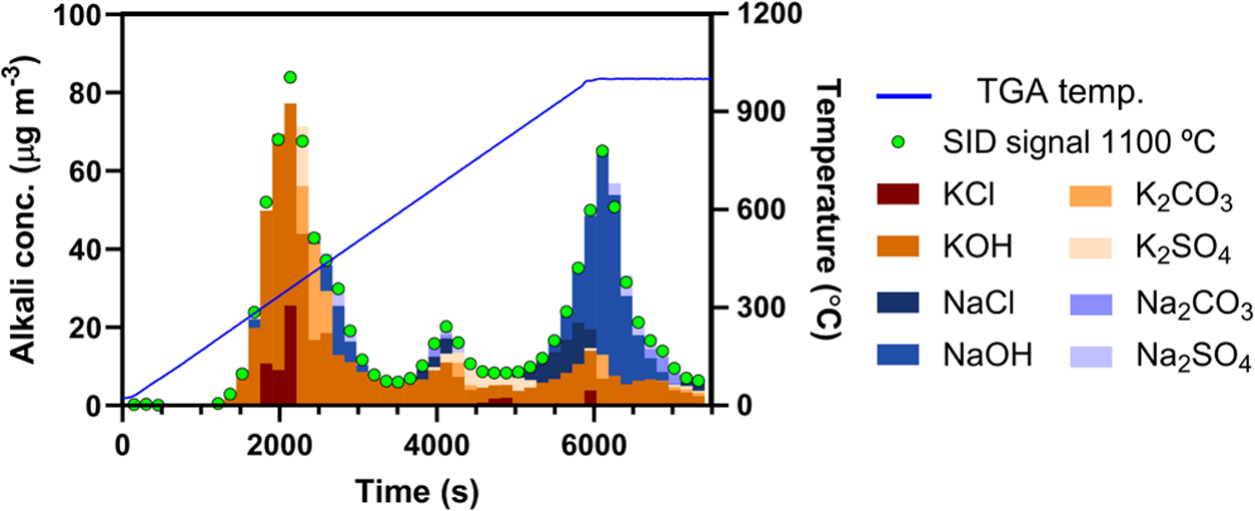
Alkali signal measured by the SID at 1100 °C filament temperature (green dots) and TGA temperature (blue) during biomass conversion under inert conditions. Alkali emission composition predicted by the TMSI model (bars).

The TMSI method was also employed in the TGA-SID setup to characterize the alkali emissions during pyrolysis of biomass.^44^ A sample of pine wood was heated to 1000 °C, and it was kept at a constant temperature for an extended time. The TGA was operated under inert conditions with pure nitrogen, and alkali emissions were measured online in the exhaust gases with the TMSI method during the simultaneous monitoring of the sample mass loss. Figure 13 shows the steady-state alkali concentration measured at the 1100 °C SID filament temperature (green dots) together with the predicted composition of the alkali emissions and the TGA temperature.

Alkali is released in two main stages, the first between 300 and 500 °C where the wood is pyrolyzed and a second when the temperature exceeds 850 °C, corresponding to alkali release from remaining char and ash. TMSI model predictions show that the alkali emissions during the pyrolysis stage are dominated by potassium compounds, mainly KOH and, to a lesser extent KCl. A minor emission of NaOH is also identified during the later stage of the pyrolysis process. The potassium compounds continue to dominate as the sample temperature increases above 550 °C. At temperatures above 700 °C, emissions of NaOH and, to a minor extent, NaCl become increasingly important and dominate over the KOH and KCl emissions at 1000 °C.

Averaged over the whole experiment, alkali emissions consist of an approximately 3:1 ratio of K and Na compounds. By integrating the alkali signal, around 0.010 wt % K and 0.003 wt % Na are released from the biomass sample.^44^ This is about a third of the available alkali and agrees well with the ratio between K and Na in the biomass sample.^67^

### 3.5 Relevance to Industrial Applications

In a chemical looping system, there is a distinct separation between when the bed material experiences reducing or oxidizing conditions. This will not be the case in a conventional fluidized bed boiler. Instead, the bed will undergo local, stochastic, and much faster shifts between reducing and oxidizing conditions. The role of alkali uptake and release and how it varies between reducing and oxidizing conditions will, despite their differences, be important in both types of systems. The findings of this study therefore provide valuable insights relevant to fuel conversion in fluidized beds using oxygen carriers in both conventional and CLC systems.

Alkali compounds are readily released into the gas phase during fuel conversion. Consequently, they are primarily introduced into fluidized beds under reducing conditions, either to the reducing fuel reactor in a CLC application or to the reducing environment that is locally formed during fuel conversion in a conventional application. The ability to monitor how alkali compounds are released from fuels during these processes is highly relevant for industrial applications. The TMSI technique demonstrated in this study enables real-time alkali speciation, providing a valuable tool for understanding and optimizing alkali release during fuel conversion processes. By offering high sensitivity, selectivity, and simplicity, the TMSI method allows for detailed tracking of individual alkali compounds, such as potassium or sodium chlorides, hydroxides, or sulfates under various conditions. This capability makes it particularly relevant for industries relying on low-grade biomass, waste, or coal as a fuel.

In these experiments, ilmenite demonstrated nearly complete alkali uptake under reducing conditions. In practice, this could significantly reduce the amount of alkali available in the flue gas. This aligns with prior studies highlighting ilmenite as an effective alkali scavenger.^27,28,72^ In contrast, calcium manganite and manganese oxide showed less efficient alkali capture under reducing conditions. A lower alkali concentration in the flue gas could reduce problems with, e.g., alkali-induced fouling and corrosion. In addition, having alkali-containing bed particles might be advantageous due to catalytic effects on fuel conversion.^6,10^ On the other hand, higher alkali content in the bed could also increase the risk of alkali-induced agglomeration of the bed particles.^73^

In a CLC system, alkali may be carried from the fuel reactor to the oxidizing air reactor via the oxygen carrier.^39^ The longer continuous residence times under oxidizing conditions in a CLC system may lead to greater alkali release compared with conventional fluidized bed systems. Since most of the heat is extracted from the gas stream leaving the exothermic air reactor in a CLC application,^12^ it is crucial to minimize alkali emissions from this reactor to mitigate alkali-induced corrosion. Alkali desorption experiments revealed that ilmenite releases a fraction of its absorbed alkali in oxidizing conditions at temperatures relevant to CLC operation.^48^ The alkali uptake experiments showed that ilmenite and calcium manganite were effective in absorbing alkali under oxidizing conditions, while manganese oxide showed less efficient alkali uptake.

The effective alkali uptake and limited alkali desorption seen in these experiments make ilmenite promising for biomass conversion applications in terms of mitigating high-temperature corrosion while potentially enhancing fuel conversion. Ilmenite also has high chemical stability and a low material cost.^18,28,71^ In contrast, calcium manganite and manganese oxide, though more expensive, are less efficient at managing alkali emissions but may provide advantages in fuel conversion and oxidizing efficiency.^47^ These trade-offs could make them suitable for applications where alkali emissions are less critical but where maximizing combustion efficiency is a priority.

## 4 Conclusions

This study highlights advancements in alkali monitoring techniques and laboratory-scale reactor systems to investigate the alkali behavior in high-temperature processes. Central to this work is the temperature-modulated surface ionization (TMSI) method, which determines the contributions of K^+^ and Na^+^ bound to Cl^–^, OH^–^, CO_3_^2–^, and SO_4_^2–^ to the alkali flux from different reactor systems. Traditional laboratory-scale fluidized bed reactors have been limited by significant alkali–wall interactions above and below the bed, hindering the accurate study of alkali dynamics within the bed. A new reactor was designed to minimize these wall interactions, enabling detailed studies of alkali uptake in fluidized beds.

By using this combination of a new reactor and the TMSI method, it was demonstrated that the type of bed material, injected salt compound, and gas conditions play a crucial role in alkali uptake efficiency. Among the oxygen carriers tested, ilmenite showed near-complete alkali absorption, making it superior to calcium manganite and manganese oxide. It was seen that injected sodium and potassium compounds behave similarly in the bed, where chlorides and sulfates, to a lesser extent, can readily escape the bed while hydroxides are mostly retained. Emission analysis with TMSI revealed that alkali chlorides and sulfates remained stable during experiments, whereas hydroxides readily formed secondary alkali compounds in the reactor.

This work also shows that it is possible to measure alkali emissions and mass loss from fixed bed samples by combining TMSI and thermogravimetric analysis (TGA), a combination that can be used to study alkali release from both fuel and bed particles. The experiments show that ilmenite samples used for biomass combustion release alkali in both inert and oxidizing environments at high temperatures. Conversion of pine wood showed emission of KOH, and to a lesser extent KCl, during the pyrolysis process, with an increasing extent of NaOH and NaCl emissions as the temperature approaches 1000 °C.

Overall, this work highlights the complexity of alkali behavior in high-temperature processes, influenced by factors including the type of alkali compounds, bed material, and gas environments. The novel monitoring techniques and reactor designs presented here provide innovative information about different alkali compounds, their interactions with fluidized beds, and their release characteristics from biomass. Monitoring alkali uptake and release in fluidized beds is important to enhance fuel conversion while also mitigating alkali-induced fouling and corrosion and limiting expensive bed replacement. Beyond fluidized beds, alkali emission speciation is highly relevant for all types of fuel conversion processes. The alkali speciation analysis could be improved further by increasing the time resolution of the TMSI method to capture alkali emission processes with rapid changes in composition or concentration.

## Supporting Information Available

Result tables that summarize the average values and error limits of alkali outlet concentrations in reducing and oxidizing conditions for all combinations of alkali salt injections and fluidized bed materials; tables representing the graphical bar plots in Figure 9 (PDF)
