# Supplementary material for: Alkali Uptake, Release, and Speciation in Fluidized Beds Using Oxygen Carriers
Source: Energy Fuels. 2025 Feb 4;39(6):3280–94. doi: 10.1021/acs.energyfuels.4c05523 (PMC11833681; doi:10.1021/acs.energyfuels.4c05523)
Supplement: Supplementary file 1 — ef4c05523_si_001.pdf [file ef4c05523_si_001.pdf]

Supporting Information

Alkali Uptake, Release and Speciation in Fluidized Beds using Oxygen Carriers

Viktor Andersson[1], Jan B.C. Pettersson[2], Thomas Allgurén[1], Pavleta Knutsson[3], and Klas Andersson[1,4]*

[1]Department of Space, Earth and Environment, Division of Energy Technology, Chalmers University of Technology, Hörsalsvägen 7A, SE-412 96 Gothenburg, Sweden
[2]Department of Chemistry and Molecular Biology, Division of Atmospheric Science, University of Gothenburg, Medicinaregatan 7B, SE-413 90 Gothenburg, Sweden
[3]Department of Chemistry and Chemical Engineering, Chalmers University of Technology, Kemigården 4, SE-412 96 Gothenburg, Sweden
[4]Department of Chemical Engineering, University of Utah, Salt Lake City, Utah 84112 United States
*Corresponding author: viktand@chalmers.se

Average alkali concentrations in reducing and oxidizing conditions

Tables S1-S3 summarizes the average values of alkali outlet concentrations in reducing and oxidizing conditions for all combinations of alkali salt injections and fluidized bed materials. Included errors indicate how much the average values deviate from the highest and lowest values of the stable alkali concentration obtained in each gas condition.

Table S1: Average outlet alkali concentrations (mg m^{-3}) in reducing and oxidizing conditions when the reactor is filled with 40 g of Mn_3O_4 particles. Positive and negative errors indicate how much the average values deviate from the highest and lowest values of the stable alkali concentration obtained in each gas condition (mg m^{-3}). The results are graphically presented in Figure 9.

	**Mn_3O_4 reducing conditions**			**Mn_3O_4 oxidizing conditions**		
	Average	Positive error	Negative error	Average	Positive error	Negative error
KCl	1.86	0.24	0.36	3.19	3.01	1.29
NaCl	5.43	0.67	1.03	7.91	8.89	3.71
KOH	0.15	0.01	0.02	0.14	0.01	0.02
NaOH	0.18	0.07	0.01	0.16	0.01	0.03
K_2SO_4	1.76	0.74	0.26	1.24	0.26	0.19
Na_2SO_4	1.56	0.24	0.18	1.13	0.27	0.23

Table S2: Average outlet alkali concentrations (mg m^{-3}) in reducing and oxidizing conditions when the reactor is filled with 40 g of $CaMnO_3$ particles. Positive and negative errors indicate how much the average values deviate from the highest and lowest values of the stable alkali concentration obtained in each gas condition (mg m^{-3}). The results are graphically presented in Figure 9.

	**$CaMnO_3$ reducing conditions**			**$CaMnO_3$ oxidizing conditions**		
	Average	Positive error	Negative error	Average	Positive error	Negative error
KCl	3.74	1.06	0.84	1.42	0.33	0.42
NaCl	2.93	1.17	0.63	1.19	0.51	0.29
KOH	0.87	0.35	0.24	0.26	0.04	0.09
NaOH	0.36	0.18	0.10	0.14	0.02	0.03
K_2SO_4	0.44	0.08	0.06	0.17	0.05	0.06
Na_2SO_4	0.9	0.15	0.13	0.58	0.22	0.18

Table S3: Average outlet alkali concentrations (mg m^{-3}) in reducing and oxidizing conditions when the reactor is filled with 40 g of ilmenite particles. Positive and negative errors indicate how much the average values deviate from the highest and lowest values of the stable alkali concentration obtained in each gas condition (mg m^{-3}). The results are graphically presented in Figure 9.

	**Ilmenite reducing conditions**			**Ilmenite oxidizing conditions**		
	Average	Positive error	Negative error	Average	Positive error	Negative error
KCl	0.25	0.12	0.06	0.56	0.39	0.29
NaCl	0.42	0.20	0.12	1.04	0.71	0.49
KOH	0.05	0.02	0.01	0.04	0.02	0.02
NaOH	0.10	0.04	0.02	0.08	0.04	0.03
K_2SO_4	0.69	0.23	0.20	0.86	0.14	0.41
Na_2SO_4	0.44	0.32	0.20	0.32	0.19	0.10
